# Evidence for the spread of the alien species *Aedes koreicus* in the Lombardy region, Italy

**DOI:** 10.1186/s13071-021-05031-7

**Published:** 2021-10-14

**Authors:** Agata Negri, Irene Arnoldi, Matteo Brilli, Claudio Bandi, Paolo Gabrieli, Sara Epis

**Affiliations:** 1 Department of Biosciences and Pediatric Clinical Research Center “Romeo Ed Enrica Invernizzi”, University of Milan, 20133 Milan, Italy; 2 Department of Biology and Biotechnology, University of Pavia, 27100 Pavia, Italy; 3 University School of Advanced Studies Pavia, IUSS, 27100 Pavia, Italy; 4 Italian Malaria Network, Inter University Center for Malaria Research, University of Milan, 20133 Milan, Italy

**Keywords:** Alien species, *Aedes koreicus*, Invasive mosquitoes, Morphological and molecular identification

## Abstract

**Background:**

*Aedes koreicus* is a mosquito species characterized by marked anthropophilic behavior, and a potential vector of nematodes and viruses. It is native to East Asia, but its presence has recently been reported in many regions of Europe. In Italy, these mosquitoes had been detected in the northeast since 2011 and are now spreading towards the southwest of the country.

**Methods:**

In 2020, during a surveillance program for invasive mosquito species in the district of Bergamo (Lombardy Region, Italy), about 6000 mosquito larvae were collected. Emerged adults were assigned to mosquito species according to morphological analyses, followed by amplification and sequencing of genetic markers (*COI*, *ND4*, *ITS2* and *D2*).

**Results:**

According to the morphological and genetic data, about 50 individuals belonged to the species *Ae. koreicus*.

**Conclusion:**

We report the presence of *Ae. koreicus* in the district of Bergamo, which confirms the spread of this species in the north of Italy and raises concerns about its possible role as a vector of diseases in the Alpine area.

**Graphical Abstract:**

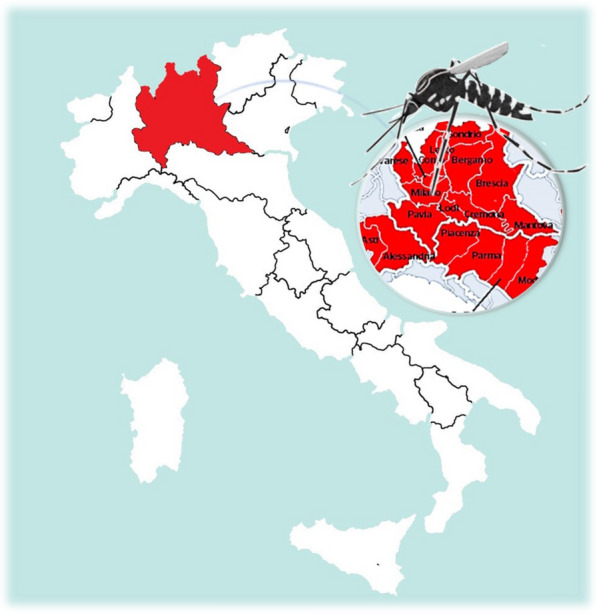

**Supplementary Information:**

The online version contains supplementary material available at 10.1186/s13071-021-05031-7.

In recent decades, the spread of alien mosquito species, as potential vectors of pathogens of public health importance, has raised serious concern in Europe [1, 2]. In addition to the well-known tiger mosquito *Aedes albopictus*, another species of this genus, *Ae. koreicus*, is spreading in Europe [2, 3]. This species is native to East Asia (Japan, Korea, China and Russia) [4] and was reported in Europe for the first time in Belgium in 2008, in the industrial zone of Maasmechelen [5]. Subsequently, individuals from this species have been repeatedly collected, starting from 2011 in Belluno and in the northeast mountainous region of Italy [6–10], and then in 2012 and 2013 at the Swiss-Italian border (Chiasso and Como) [11], in 2013 in a village in northeastern Slovenia [12], in 2015 in southern Germany [13] and Genoa (Italy) [14], in 2016 in central Germany [15] and Pécs (Hungary) [16], and in 2018 in East Tyrol in Austria [17].

Here we report the detection of a novel population of *Ae. koreicus* in Italy, in the Bergamo district (in the Lombardy region). This novel record highlights that this species is likely expanding in Lombardy, especially in mountain areas. Identification of the collected mosquitoes was achieved by morphological observations and molecular analysis of three genetic markers, which were then also used to reconstruct phylogenetic trees. Our record further emphasizes the urgent need for coordinated efforts for the monitoring of invasive mosquitoes in Italy, to prevent the spread of vectors of emerging pathogens.

In a surveillance program for invasive mosquito species in the Bergamo district (45° 43′ 02.3″ N 9° 50′ 38.0″ E, 370 m above sea level, Lombardy Region, Italy) (Additional file 1: Figure S1), about 6000 larvae and hundreds of mosquito eggs were collected from a pond and several artificial tanks; we collected larvae and eggs during four catches, between July and September 2020 (end of July, mid-August, and the beginning and end of September). Samples were then reared in the insectarium under standard conditions (26 °C, 80% humidity). After each harvest, the larvae were separated into three groups, according to the observed morphological differences; About 4800 larvae were identified as belonging to the genus *Culex*, based on the recognition of the peculiar traits of this genus at the larval instar. Some of the remaining larvae were observed and morphologically characterized under a stereomicroscope before pupation; most of them emerged as adults and were observed for further identification. Based on the morphological identification keys described in Tanaka et al. [4], Versteirt et al. [5] and Pfitzner et al. [15], about 50 larvae and adult mosquitoes were identified as *Ae. koreicus*. The remaining mosquitoes were identified as belonging to the genus *Culiseta.*

As reported in Fig. 1, the head of *Ae. koreicus* larvae shows the four head hairs (cranial setae) lining the frontal clipeal edge and about 18 evenly spaced teeth on siphonal pecten. Moreover, adult mosquitoes of *Ae. koreicus* show a pale basal band at the hind tarsomeres IV and the typical scutal pattern. The base of the posterior femur is completely pale, and a subspiracular patch of pale scales is usually present. These features distinguish *Ae. koreicus* from the closely related invasive mosquito *Ae. japonicus japonicus*, which instead usually presents dark hind tarsomeres IV and a dark subbasal band on the posterior femur. Moreover, *Ae. j. japonicus* mosquitoes lack the subspiracular patch [15]. In recent years, two main populations of *Ae. koreicus* have been identified as invasive mosquitoes in Europe: the Korean mainland population and the South Korean volcanic island Jeju population [4, 5]. The two populations can be partially distinguished by morphological analysis, since the Korean mainland population is characterized by a usually dark hind tarsomere V [4]. On the contrary, the Jeju population presents, in the same body part, a pale basal band, a characteristic also observed in *Ae. koreicus* mosquitoes collected and maintained in our insectarium. Thus, we could attribute the individuals of *Ae. koreicus* collected in Lombardy to the Jeju morphological variant, which was previously reported in Belgium [5] and in the Veneto region in Italy [6].

**Fig. 1:**
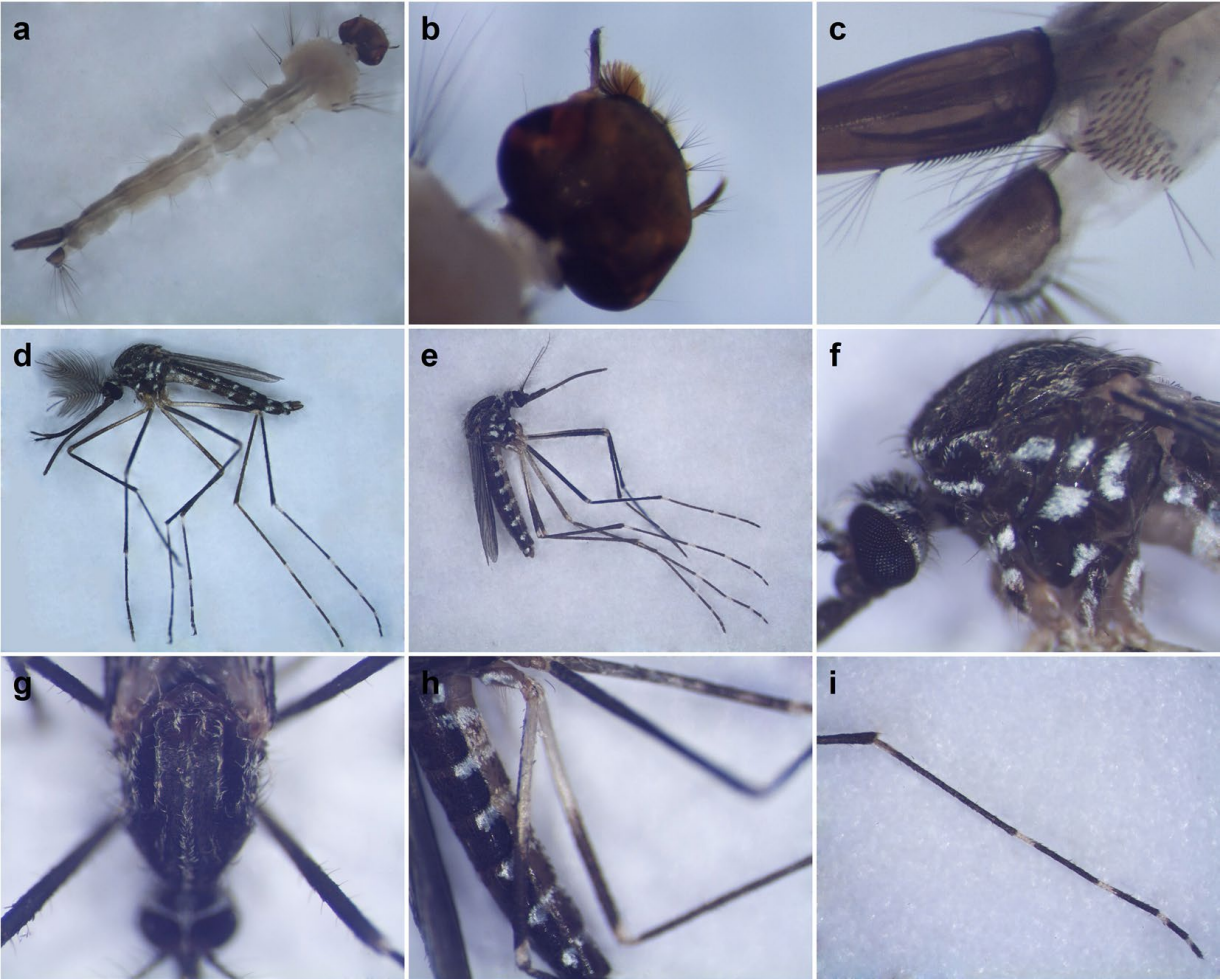
Morphological features of *Aedes koreicus* larvae (**a**–**c**) and adult mosquitoes (**d**–**i**). The fourth-instar larva (**a**) carries setae with multiple branches in the frontal region of the head (**b**), while about 18 evenly spaced teeth are located on the siphonal pecten (**c**). Male (**d**) and female (**e**) adults show typical sexual dimorphism. The subspiracular area has a distinct patch of pale scales (**f**); the dark mesonotum harbors five defined lines forming a peculiar pattern with yellowish-brown or golden-yellow scales (**g**); the posterior femurs are characterized by a completely pale base (**h**), and the hind tarsomere IV shows the typical pale basal band (**i**)

To confirm the morphological identification, molecular analyses were performed on adult female mosquitoes. Methods for molecular identification of mosquito species, such as the one developed by Cameron et al. (2010) [18], are useful for the identification of field-collected specimens. Indeed, morphological identification of *Ae. koreicus* can be misleading because of peculiar features partially overlapping with those of *Ae. japonicus* subspecies. Moreover, samples can be damaged: from our experience, some morphological characters, such as the scales forming the design of the mesonotum, can be lost if mosquitoes are stored for a long time, making the identification difficult. In this study, following polymerase chain reaction (PCR)-based identification as described in Cameron et al. [18], the sequencing of four loci (*COI*, *D2*, *ND4* and *ITS*) allowed us to further confirm the identification of the collected samples and to reconstruct the phylogenetic relationship between *Ae. koreicus* and other mosquito species. DNA was extracted from whole bodies of individual mosquitoes (Monarch^®^ Genomic DNA Purification Kit, New England Biolabs). A first molecular analysis was performed following Cameron et al. [18]: the consistent presence of a single-nucleotide polymorphism (SNP) in the nicotinamide adenine dinucleotide dehydrogenase subunit 4 (*ND4*) gene of *Ae. koreicus* has enabled the development of a PCR tool for rapid identification of this species. This PCR protocol uses the *Ae. koreicus*-specific primer ND4korF (5′-CCCCATTTAACCCCCAATAT-3′), together with the primers N4J8502D(F) and N4N-8944D(R), to amplify a single band of 465 bp in the control species *Ae. albopictus* and an additional band of 283 bp for *Ae. koreicus* (Additional file 2: Figure S2). This protocol confirmed the identification as *Ae. koreicus* for all individuals previously subjected to morphological observations.

Four other molecular markers were amplified and sequenced: two mitochondrial loci, cytochrome oxidase I (*COI*) and *ND4*, and two nuclear (ribosomal) loci, the internal transcribed spacer 2 (*ITS2*) and the gene portion coding for the 28S ribosomal subunit 2 (*D2*). To amplify these DNA fragments, specific primers and protocols, already published, were followed, as reported in the Additional file 3: Table S1.

The sequences obtained from the PCR products (Eurofins Genomics, Ebersberg, Germany) were used to build phylogenetic trees including sequences from closely related species, retrieved using the nucleotide Basic Local Alignment Search Tool (BLAST) [19]. Alignments were obtained using MUSCLE (MUltiple Sequence Comparison by Log-Expectation) [20], with default parameters for nucleotide sequences. After manual editing, we ran PhyML (phylogenetic maximum likelihood ) [21, 22] with automatic selection [23] of the best phylogenetic model for the data and 1000 bootstraps. For all three genes analyzed in this study, the best model turned out to be GTR+G+I+F, and the corresponding trees are shown in Fig. 2.

**Fig. 2:**
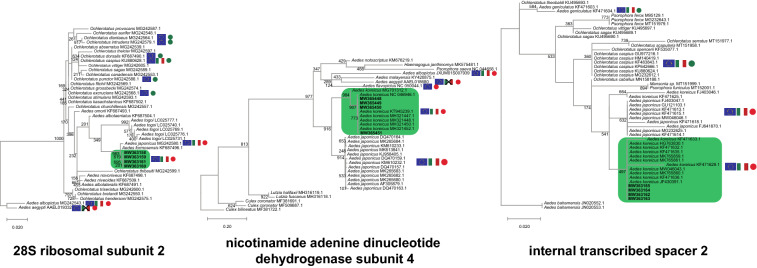
Phylogenetic trees for *D2*, *ND4*, *ITS* sequence data. Trees were built using PhyML, with automatic model selection, gamma distribution rates (discretized using four categories) and an estimated proportion of invariant sites. Node support was obtained by running 1000 bootstrap replicates. Models selected by SMS (Smart Model Selection) were HKY85 (*ND4*), K80 (*ITS*) and GTR (*D2*). Mosquito species found in Europe, and particularly in Italy, are tagged with the respective national flag. *Aedes aegypti*, *Ae. albopictus*, *Ae. japonicus* and *Ae. koreicus* are invasive species (red point), while *Ochlerotatus diantaeus*, *Och. intrudens*, *Och. dorsalis*, *Och. caspius*, *Och. punctor* and *Och. excrucians* are native species (green point)

Because of the lack of enough sequences of related species in GenBank, no tree was constructed for the *COI* gene. However, we obtained a sequence for *Ae. koreicus* that showed 98–100% identity with those in the databases. This sequence and the other three were all deposited in GenBank (MW365452, MW365453, MW365454, MW365455).

The sequences obtained from the amplification of the *ND4* region (MW365448, MW365449, MW365450) are 100% identical to the *Ae. koreicus* sequences from Germany (GenBank: KT945239.1) and Italy (GenBank: MH321447.1, MH321448.1, MH321450.1, MH321452.1), while only a single nucleotide in the sequence MW365451 was different. As reported in Fig. 2, the *ND4* phylogenetic tree highlighted that our sequences clustered with a Korean *ND4* sequence (from the mitochondrial genome deposited in GenBank NC 046946.1) with 90.7% bootstrap support. As expected, the *Ae. koreicus* monophylum clustered with *Ae. japonicus* sequences with 91.6% bootstrap support. In fact, according to the phylogenetic analysis of Cameron et al. [18], *Ae. koreicus* forms a monophyletic group with the four *Ae. japonicus* subspecies.

Figure 2 reports also the phylogenetic tree constructed starting from the *D2* nuclear region sequences (GenBank: MW363158, MW363159, MW363160, MW363161): our sequences clustered with an *Ae. formosensis* sequence (GenBank: KF687496.1) and an *Ae. japonicus* sequence (GenBank: MG242580.1) with 98.3% bootstrap support. Indeed, the three species are recognized members of the *Hulecoeteomyia* subgenus [24].

All the four sequences of the *ITS2* region (GenBank: MW363162, MW363163, MW363164, MW363165) were identical to *Ae. koreicus* sequences from Russia, Belgium and Hungary (GenBank: MK765860.1, KF471636.1, JF430391.1, MW046043.1, HG763830.1).

Our results confirm the high genetic similarity between *Ae. koreicus*, *Ae. japonicus* and the other species belonging to the *Hulecoeteomyia* subgenus [4].

All these analyses confirm the presence of *Ae. koreicus* in the study area. This species is an Asian mosquito species native to Korea, Japan, China and Eastern Russia, which has become increasingly diffused in Europe [11, 12, 16]. Members of this species show great adaptability to temperate regions, probably because *Ae. koreicus* tolerates low temperatures and prefers mountain regions (23–28 °C) [25]; furthermore, adults of *Ae. koreicus* have greater persistence during the late summer and autumn seasons [26].

In Italy, *Ae. koreicus* has colonized an area of more than 3000 km^2^ in the hills and pre-Alpine zones (from Liguria to Trentino and Veneto regions). The spread of *Ae. koreicus* mosquitoes is rapidly increasing towards southern and western Italy, from the original infested area in the district of Belluno, probably due to the presence of dense road connections and suitable habitats in other areas [8]. The ability to colonize areas characterized by harsh winter temperatures allows *Ae. koreicus* mosquitoes to avoid competition for breeding sites occupied by species that have demonstrated a considerable advantage in terms of number and speed of replication, such as *Ae. albopictus* [26]. In this regard, no specimens of *Ae. albopictus* were found at the collection site of *Ae. koreicus* near Bergamo. Larval competition between *Ae. albopictus* and *Ae. koreicus* under natural conditions needs to be thoroughly studied and should be integrated with investigations on the interspecific interactions with other species such as *Culex* spp. and *Culiseta* spp., found at the same breeding site of *Ae. koreicus.*

The finding of *Ae. koreicus* in the pre-Alpine area in the Bergamo district, particularly at the end of the summer season, is a further confirmation of the adaption of these mosquitoes to specific climate and environmental conditions; our georeferenced record will thus contribute to the prediction of future expansion patterns for this species. The distribution area of *Ae. koreicus* is indeed widening, under the influence of global warming and the heavier international trade and travels in Europe [27]. Additionally, several laboratory experiments suggest that *Ae. koreicus* is a potential vector of pathogens, such as the nematodes *Dirofilaria immitis* and *Brugia malayi* [28] and viruses that cause chikungunya [29] and Japanese encephalitis [30]. The identification of *Ae. koreicus* during a sampling that was not focused on the collection of *Aedes* mosquitoes highlights the importance of the monitoring activity and the need for wider knowledge of the introduction routes and the ecological niches occupied by this invasive mosquito. The clarification of some aspects of the biology of the Korean mosquito, such as biting habits, together with a deeper investigation of its vector status, will be fundamental to evaluate the risk of the spread of *Ae. koreicus* for public health in Europe.

The morphological features of the population of *Ae. koreicus* mosquitoes caught in the Bergamo district are comparable to those typical of the Jeju Island population, from which the majority of European populations seem to derive. This observation raises questions about the introduction and expansion of this alien species in Italy and, more broadly, in Europe. One plausible way of introduction in the Alpine area of Lombardy is the exchange of goods and people occurring in the international airport of Orio al Serio, in the Bergamo district. The presence of an international airport was also suggested as the possible importation route to the Liguria region [14]. Alternatively, *Ae. koreicus* could have been introduced in the Bergamo area from other infested areas in Italy. It must be noted that the road transport in this region is very intense, and previous surveys have reported the presence of the species in the nearby districts of Vicenza and Verona, in the Veneto region [8]. Dispersal models predict that parts of the Po and Adige valleys will be colonized by *Ae. koreicus* in the next decade due to the presence of trade roads [31]. Since control measures to eradicate this species have not yet been applied, an accurate means of monitoring might allow us to reconstruct and follow the dispersal routes of this alien species, and thus to plan targeted interventions aimed at the containment of its spread.

In conclusion, we report the presence of *Ae. koreicus* in the district of Bergamo, which confirms the rapid spread of this species in this area and raises concerns about its possible role as a vector of diseases in the Alpine area.

## Supplementary Information

**Additional file 1: Figure S1.** Localization of the collection site of *Aedes koreicus* mosquitoes in the Bergamo district (Lombardy Region, Italy) on Google Earth.

**Additional file 2: Figure S2.** Agarose gel electrophoresis showing results of *Aedes koreicus* assay with N4J8502D(F), N4N-8944D(R) and ND4korF primers. *Ae. albopictus* specimen displayed a single fragment of 465 bp (code number 1). *Ae. koreicus* specimens produced two fragments, one common fragment of 465 bp and the species-specific fragment of 283 bp (code number 3–7). M molecular weight standard (BenchTop 100 bp DNA Ladder, PROMEGA).

**Additional file 3: Table S1.** Primer sequences and references used for *Aedes koreicus* identification. PCRs were performed for two mitochondrial loci, cytochrome oxidase I (*COI*) and nicotinamide adenine dinucleotide dehydrogenase subunit 4 (*ND4*), and for two nuclear (ribosomal) loci, the internal transcribed spacer 2 (*ITS2*) and the 28S ribosomal subunit 2 (*D2*).

## Data Availability

The nuclear and mitochondrial DNA sequences obtained in this study were deposited in the GenBank database under the accession numbers MW365452-MW365455 for the *COI* gene, MW365448-MW365451 for the *ND4* region, MW363158-MW363161 for the *D2* region and MW363162-MW363165 for the *ITS2* region.

## Abbreviations

*COI*: Cytochrome oxidase I
*ND4*: Nicotinamide adenine dinucleotide dehydrogenase subunit 4
*ITS2*: Internal transcribed spacer 2
*D2*: 28S ribosomal subunit 2
PCR: Polymerase chain reaction
SNP: Single-nucleotide polymorphism
BLAST: Basic Local Alignment Search Tool
PhyML: Phylogenetic Maximum Likelihood
SMS: Smart Model Selection
