# Supplementary figures and images for: Evidence for the spread of the alien species Aedes koreicus in the Lombardy region, Italy
Source: Parasit Vectors. 2021 Oct 14;14:534. doi: 10.1186/s13071-021-05031-7 (PMC8515701; doi:10.1186/s13071-021-05031-7)

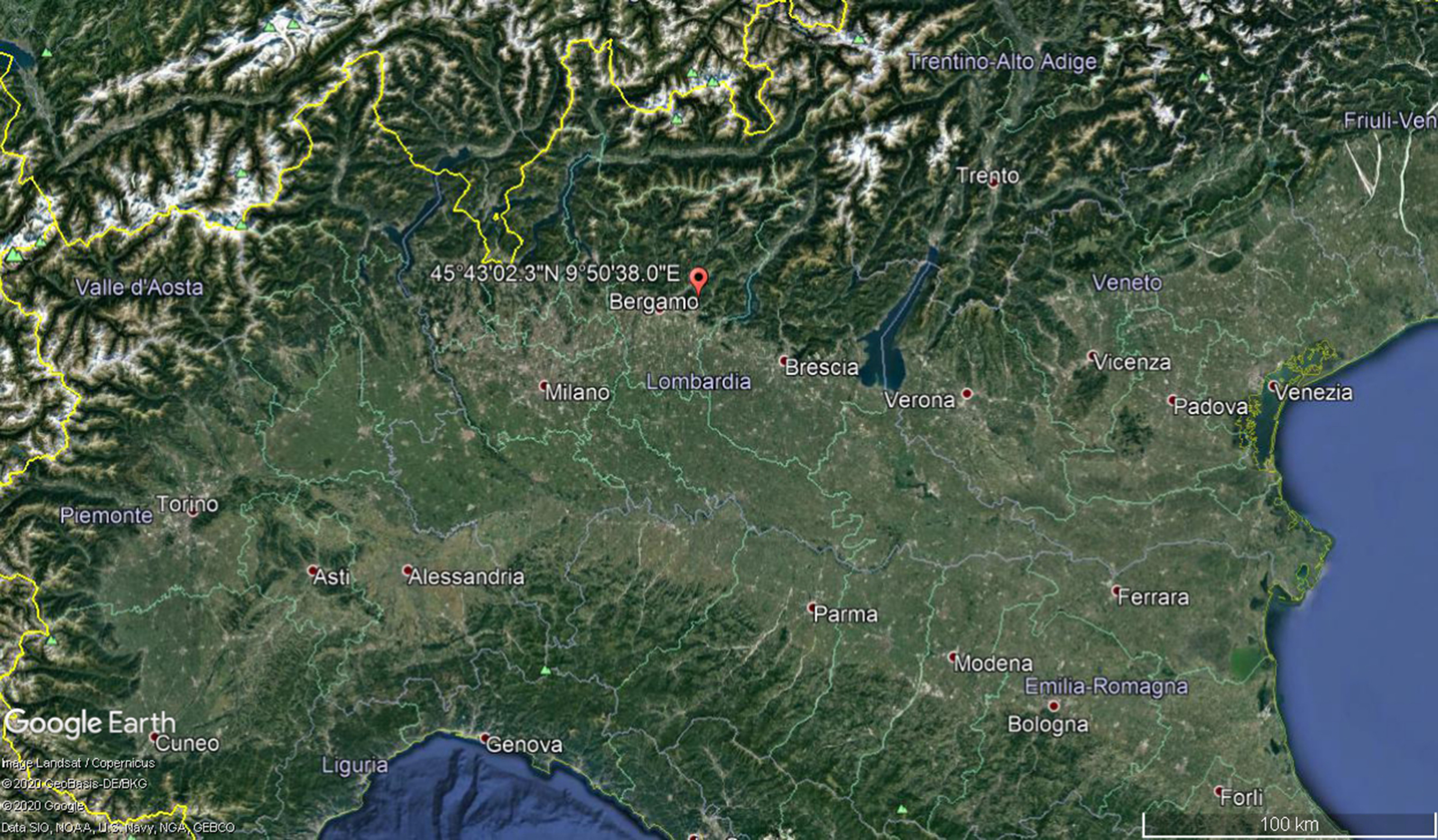

Supplement: Supplementary file 1 — Additional file 1: Figure S1. Localization of the collection site of Aedes koreicus mosquitoes in the Bergamo district (Lombardy Region, Italy) on Google Earth. [file 13071_2021_5031_MOESM1_ESM.jpg]

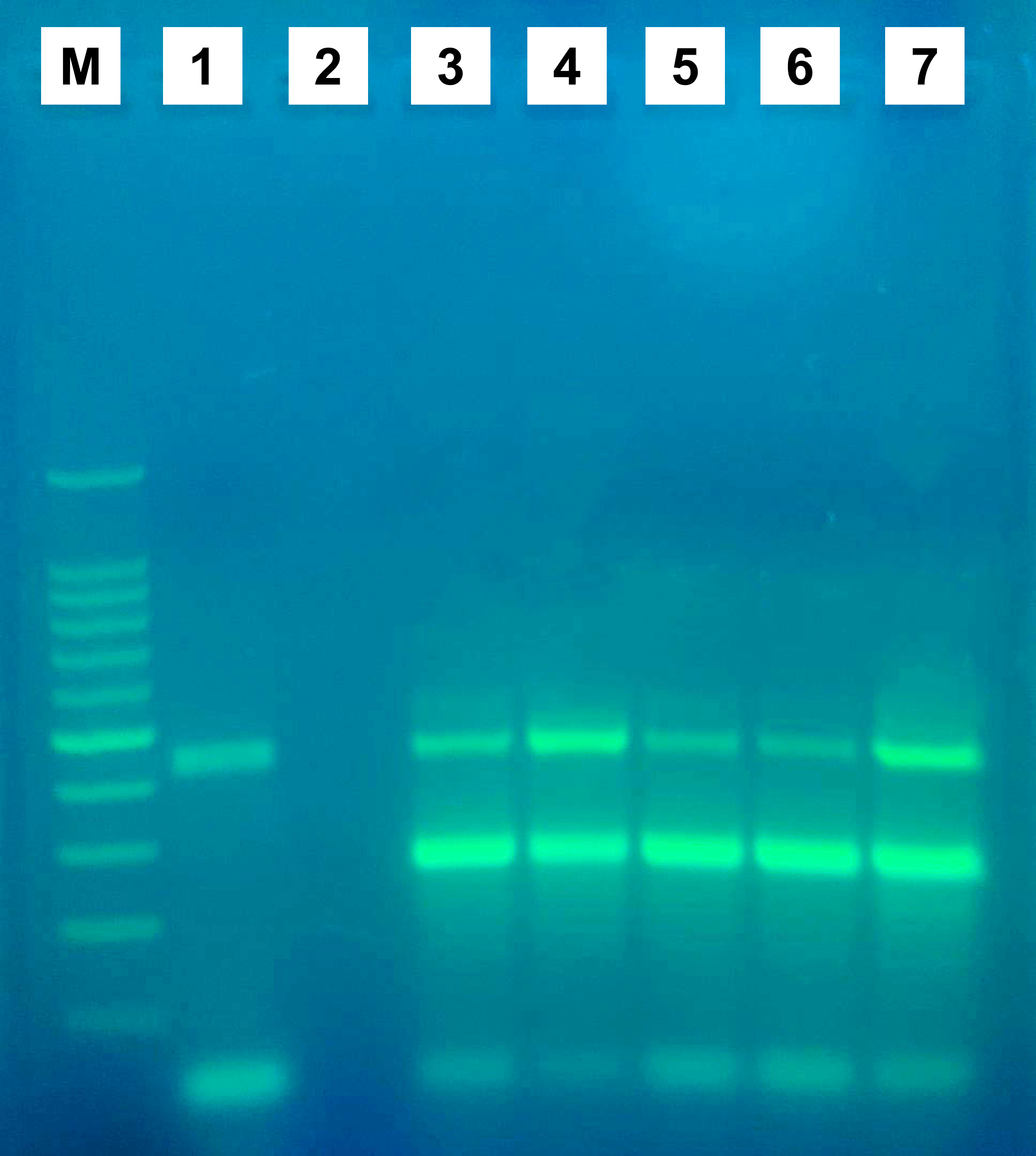

Supplement: Supplementary file 2 — Additional file 2: Figure S2. Agarose gel electrophoresis showing results of Aedes koreicus assay with N4J8502D(F), N4N-8944D(R) and ND4korF primers. Ae. albopictus specimen displayed a single fragment of 465 bp (code number 1). Ae. koreicus specimens produced two fragments, one common fragment of 465 bp and the species-specific fragment of 283 bp (code number 3–7). M molecular weight standard (BenchTop 100 bp DNA Ladder, PROMEGA). [file 13071_2021_5031_MOESM2_ESM.png]
