# Supplementary material for: Evidence for the spread of the alien species Aedes koreicus in the Lombardy region, Italy
Source: Parasit Vectors. 2021 Oct 14;14:534. doi: 10.1186/s13071-021-05031-7 (PMC8515701; doi:10.1186/s13071-021-05031-7)
Supplement: Supplementary file 3 — Additional file 3: Table S1. Primer sequences and references used for Aedes koreicus identification. PCRs were performed for two mitochondrial loci, cytochrome oxidase I (COI) and nicotinamide adenine dinucleotide dehydrogenase subunit 4 (ND4), and for two nuclear (ribosomal) loci, the internal transcribed spacer 2 (ITS2) and the 28S ribosomal subunit 2 (D2). [file 13071_2021_5031_MOESM3_ESM.docx]

| **Gene** | **Forward primer** | **Reverse primer** | **Product** | **References** |
| --- | --- | --- | --- | --- |
| ITS2 | 5,8S  5'-TGTGAACTGCAGACGACATG-3’ | 28S  5'-ATGCTTAAATTGGGGGGTA-3’ | ~370bp | Collins et al, 1996 [1] |
| D2 | D2F 5′-AGTCGTGTGTTGCTGGATAGTG-3′ | D2R  5′-CTTGGGCCGTCCGTGTTCAAGAGAG-3′ | 480 bp | Sallum et al., 2002 [2] |
| ND4 | N4J-8502D  5’-CGTAGGAGGAGCAGCTATATATT-3’ ND4korF  5’-CCCCATTTAACCCCCAATAT-3’ | N4N-8944D 5-AAGGCTCATGTTGAAGCTCC-3’ | 465 bp  283 bp | Fonseca et al., 2001 [3] |
| COI | LCO1490 5'-GGTCAACAAATCATAAAGATATTGG-3' | HC02198 5'-TAAACTTCAGGGGTGACCAAAAATCA-3' | 648 bp | Folmer et al., 1994 [4] |

1. Collins FH, Paskewitz SM. A review of the use of ribosomal DNA (rDNA) to differentiate among cryptic *Anopheles* species. Insect Molecular Biology. 1996;5(1):1–9.

2. Sallum MAM, Bergo ES, Flores DC, Forattini OP. Systematic studies on *Anopheles galvaoi* Causey, Deane & Deane from the subgenus *Nysssorhynchus* blanchard (Diptera: Culicidae). Mem Inst Oswaldo Cruz. 2002;97(8):1177–89.

3. Fonseca DM, Campbell S, Crans WJ, Mogi M, Miyagi I, Toma T, et al. *Aedes* (*Finlaya*) *japonicus* (Diptera: Culicidae), a newly recognized mosquito in the United States: analyses of genetic variation in the United States and putative source populations. J Med Entomol. 2001;38(2):135–46.

4. Folmer O, Black M, Hoeh W, Lutz R, Vrijenhoek R. DNA primers for amplification of mitochondrial cytochrome c oxidase subunit I from diverse metazoan invertebrates. Mol Mar Biol Biotechnol. 1994;3(5):294–9.
